# The Influence of a Health Promotion Program on Health and Paid Employment Among Long-Term Non-employed Individuals in the Netherlands

**DOI:** 10.1007/s10926-025-10290-7

**Published:** 2025-05-09

**Authors:** Roos W. Hijdra, Marike van Kalken, Stijn de Zeeuw, Arie Dijkstra, Alex Burdorf, Merel Schuring

**Affiliations:** 1 Department of Public Health, Erasmus University Medical Center Rotterdam, PO Box 2040, 3000 CA Rotterdam, The Netherlands; 2 Bewegen Werkt, Plesmanweg 9C, 7602 PD Almelo, The Netherlands; 3 Faculty of Behavioural and Social Sciences, University of Groningen, Grote Kruisstraat 2/1, 9712 TS Groningen, The Netherlands

**Keywords:** Health promotion, Unemployment, Mental health, Health

## Abstract

**Purpose:**

Long-term unemployment is accompanied by worse health, making it challenging to enter paid employment. This study aims to investigate effects of a health promotion program on physical and mental health, work ability, and entering paid employment among long-term non-employed individuals.

**Methods:**

In a longitudinal study, Exercise Works participants (*N* = 208) and a treatment-as-usual group (*N* = 117) were followed with measurements at baseline, 3 months, and 12 months. The Exercise Works program is a health promotion program that lasts 12 to 18 weeks. It consists of individual- and group-based physical exercises, lifestyle education, and individual coaching for two half days per week. A generalized linear mixed model for repeated measurements was used to investigate changes within individuals in health status, employment participation, and work ability during the Exercise Works program in comparison to the control group. Subgroup analyses were performed based on socio-demographic characteristics and a per protocol analysis. Interviews with 20 participants and 21 professionals were conducted.

**Results:**

This study demonstrated no significant improvements in physical and mental health, work ability and being in paid employment participation among participants of Exercise Works compared to the control group. Participants and professionals had a very positive impression of the Exercise Works program.

**Conclusion:**

Despite the Exercise Works program being positively received, the effect evaluation did not demonstrate its effectiveness. Complex problems of non-employed persons should be addressed when developing a health promotion program.

**Supplementary Information:**

The online version contains supplementary material available at 10.1007/s10926-025-10290-7.

## Introduction

In the Netherlands, half of all social benefits recipients have been unemployed for at least 5 years. While the total number of social benefits recipients is decreasing, the number of long-term recipients is actually increasing [1]. Particularly long-term unemployed persons have worse health and higher burden of disease compared to short-term unemployed persons. Such ill health encompasses the prevalence of various chronic diseases as well as decreased self-perceived physical and mental health [2–4].

Unemployed individuals face less time-structure, social contacts, social status, collective purpose, and activity [5]. This may lead to an unhealthy lifestyle due to physical inactivity, unhealthy diet, smoking or alcohol consumption [5, 6]. Dietary habits actually deteriorate as the duration of unemployment increases [7]. This can create new health problems or worsen existing health problems, as an unhealthy lifestyle is one of the major contributors to ill health [8]. However, mental health problems and financial stress may also be a barrier for adopting a healthy lifestyle. Ill health and preexisting health problems may also lead to non-employment [9].

For long-term unemployed individuals, it can be challenging to enter paid employment. Health problems are often a barrier to finding paid employment. In addition, individuals with health problems may perform less active job search behavior due to decreased psychosocial factors such as self-esteem or mastery [10]. However, even with ill health, maintaining a positive attitude toward job-searching and one’s professional abilities will enhance the likelihood of finding paid employment [10, 11]. Lastly, goal setting and being physically active affect job search behavior positively [11].

Since employment and health are interrelated, it can be beneficial to promote health and employment simultaneously [12, 13]. Therefore, the Exercise Works (Dutch: Bewegen Werkt) program was offered to social benefits recipients by the employment services. Exercise Works is a health promotion program for non-employed individuals consisting of physical activity and lifestyle education in a group setting and individual coaching. An Exercise Works coach works closely together with the employment services. The program trains employment skills, social skills, self-esteem, attitude, and intentions for healthy lifestyle behaviors throughout the program in order to guide individuals to a volunteering job or (paid/subsidized) employment.

The present target group consists of people who are unemployed despite the tight labor market and the various incentives of employment. Therefore, they can be regarded as a hardcore group that needs special attention and special measures. In addition, eligibility criteria for disability schemes have tightened in recent years due to demographic pressures, such as population aging. Consequently, many individuals who would previously qualify for disability benefits now fall under social assistance schemes, which provide only a minimal safety net.

The previous version of this program did not have beneficial effects. In a study performed in 2009, the authors described three possible explanations: the intensity of the program being too low, difficulties in the multidisciplinary collaboration of Exercise Works with the municipality to increase employment outcomes, and lack of sufficient support by the employment services after finishing the program in health promotion [14]. Therefore, improvements were made to the program to increase the effectiveness of the program. The multidisciplinary collaboration was strengthened by interdisciplinary meetings of the employment professional with the coach and the participant halfway through the program to provide support and plan a smooth transition after the program. Both employment and health related activities were explored and planned in these meetings to keep up the momentum of the Exercise Works program. Additionally, the intensity of the program was increased by investigating lengthening the program through spreading out the meetings or introducing a follow-up meeting. Behavior change messages were provided to participants throughout the week on days without program activities to increase the contact moments and motivation. Motivational interviewing techniques were adopted by the coaches.

Because the Exercise Works program has been redeveloped after previous research showed no effects and the economic context has changed, this study evaluated the redeveloped Exercise Works program. Therefore, this study aims to investigate the effects of Exercise Works on physical and mental health, employment participation, and work ability. Differences between subgroups in the effectiveness of the program, based on socio-demographic characteristics and participation rate in the Exercise Works program, will be studied. Finally, this study explores barriers and facilitators of the Exercise Works program.

## Methods

### Study Population

The study population consisted of participants of the Exercise Works program and a control group. Both groups had to be of working age (between 18 and 66 years) and not in paid employment at the start of the study. Participants of the Exercise Works program were referred to the program by an employment professional of the municipality. They were social assistance benefits recipients.

At the beginning of the study, similar inclusion criteria were in place for the control group, meaning they had to be social assistance benefits recipients as well. Due to low enrollment rates in the study, the study population was broadened to non-employed persons who lived in the Netherlands at the start of this study.

### Study Design

In this longitudinal study, the Exercise Works group was compared with a treatment-as-usual group over a 12-month period with measurements at baseline, at 3 months follow-up and at 12 months follow-up, between October 2021 and January 2024. A generalized linear mixed model for repeated measurements was used to investigate changes within individuals in health status, employment participation, and work ability during the Exercise Works program in comparison to the control group. The Medical Ethical Committee of Erasmus MC Rotterdam declared that the Medical Research Involving Human Subjects Act does not apply to the current study (MEC-2020–0771).

### Intervention: Exercise Works

The Exercise Works program is a health promotion program with a duration of 12 to 18 weeks for non-employed individuals with a large distance to the labor market. It consists of individual- and group-based physical exercises, lifestyle, and individual coaching. The program consists of two sessions per week with a duration per session between 3 and 4 h. Each session consists of visiting a gym and either going to a sports facility or a lifestyle education session. The program is taught by an educated Exercise Works coach. Physical exercise consists of visiting a gym to perform individual exercises, as well as visiting a sports hall, sports field or another type of sports facility where group-based exercises are performed in a game format. Such group-based exercises enhance skills that participants need in work and social situations, such as working together with others, compliance with rules, showing discipline and fulfilling different (social) roles. The lifestyle education lessons aim to increase knowledge on different themes (physical activity, smoking, alcohol, nutrition, sleep, stress, and relaxation) as well as to generate behavior change. Through different educational activities, this knowledge is translated to the participant’s situation. Subsequently, SMART goals on these subjects are formulated together with the Exercise Works coach. Individual coaching is performed in both formal meetings as well as in the regular Exercise Works gatherings. The Exercise Works coach uses different theoretical frameworks (e.g., GROW model, Stages of Change Model, Motivational Interviewing [15]) to change the behavior of the participant. The Exercise Works program can be adapted to the needs and preferences of the participants and the coach, as well as to the needs and facilities of different municipalities.

### Control Treatment: Usual Employment Programs

In the Netherlands, one of the requirements for receiving social security benefits is to search for a job or to participate in activities that promote employability, both offered by the employment services. They support citizens in searching for paid employment by offering different activities, programs, training, workshops or matching their clients with potential employers. This includes but is not limited to programs, such as job search or cv writing support, apprenticeships, volunteer jobs or education. In order to enroll in these programs, non-employed citizens meet about twice a year with their employment professional to create a plan for their search for paid employment. Therefore, the control group consisted of participants who were non-employed but who may have participated in different (re-)employment programs.

### Participant Recruitment

From October 2021 until January 2023, coaches who organized the Exercise Works program informed individuals who were enrolled in the Exercise Works program about this study. If the client wanted to participate, informed consent was obtained. All questionnaires were administered by the Exercise Works coaches and, after pseudonymization, forwarded to the researchers.

The control group was recruited through three different methods. Firstly, employment professionals in various municipalities asked their clients if they wanted to participate in this study. If this was the case, contact details of this client were provided to the researchers, so they could get into contact with the client (*N* = 21). Secondly, a researcher visited the employment services to ask clients in the waiting room if they wanted to participate in this study (*N* = 21). After three visits, this was no longer possible due to COVID-19. Thus, the last recruitment was conducted online in several Facebook groups (*N* = 75). An informative post about this study was placed in multiple groups where non-employed individuals were likely to be a part of (e.g., vacancies/looking for a job, free things or trading things groups). Individuals could acquire more information and enter this study through an URL link.

**Fig. 1:**
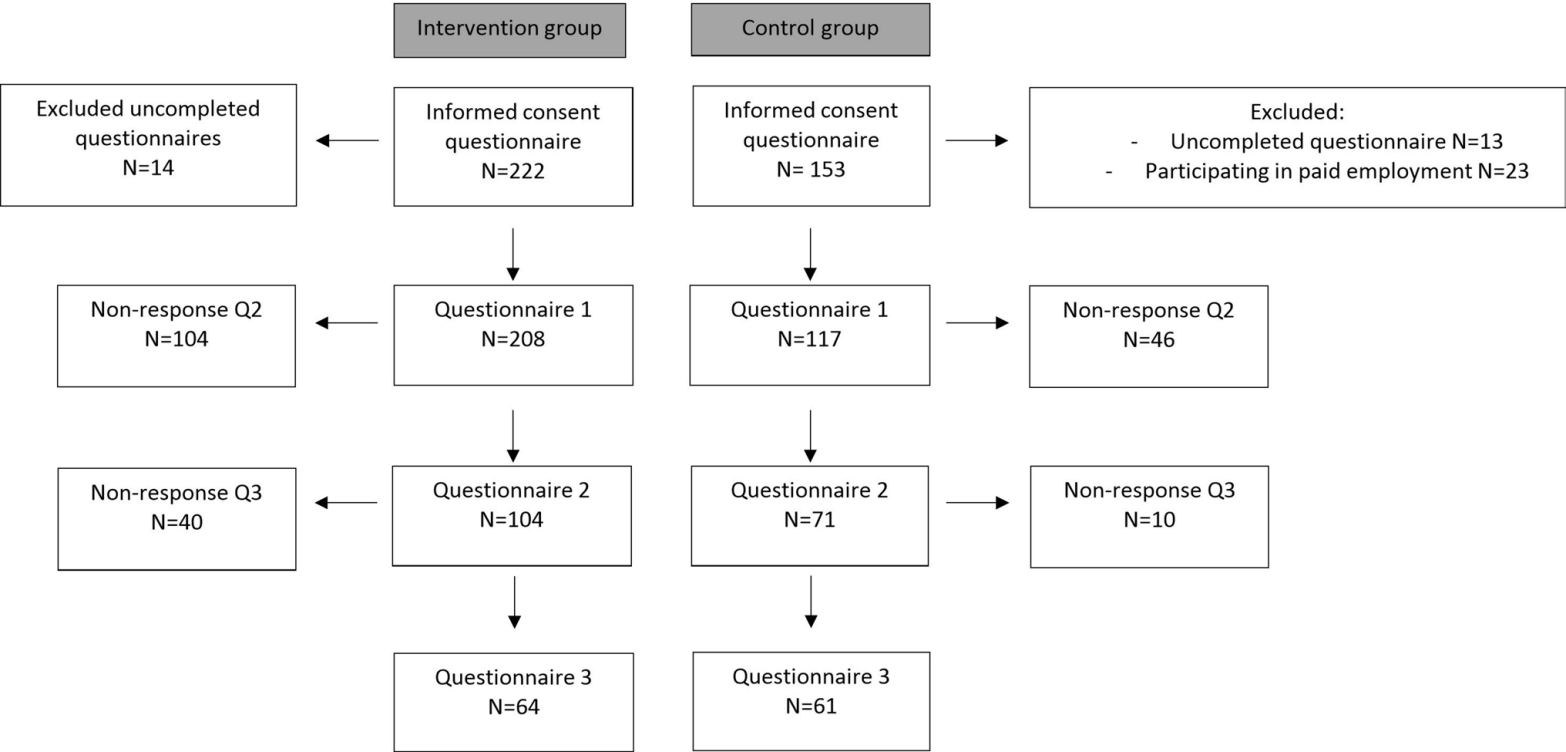
Flowchart of respondents of the exercise works program and the control group at each questionnaire 1 (baseline), questionnaire 2 (3 months), and questionnaire 3 (12 months)

Figure 1 shows the flow of participants through the study. In total, 222 participants of Exercise works and 153 participants of the control group provided informed consent to participate in this study. However, 14 Exercise Works participants and 13 control group participants were excluded due to incomplete baseline measurements. Additionally, 23 participants of the control group were participating in paid employment for more than 12 h per week and were excluded. Consequently, 208 Exercise Works participants with completed questionnaires and 117 control group participants with complete questionnaires were included. The second questionnaire (3 months) was completed by 104 Exercise works participants and 71 control group participants. The last questionnaire at 12 months was completed by 64 participants of Exercise Works and 61 participants of the control group.

### Data Collection

For the Exercise Works group, questionnaires were completed online or with the guidance of the Exercise Works coach between October 2021 and January 2024. The control group completed their questionnaires online or on paper with the available help of a researcher between November 2021 and January 2024. Reminders for the control group were sent two times, respectively, two and four weeks after sending out the questionnaire.

### Health

Perceived physical and mental health were measured with the SF-12 questionnaire. The SF-12 is a questionnaire with 12 items on general perceived health. These questions were split into two sub scales based on 6 physical health questions and 6 questions on mental health [16]. The physical and mental health subscales were used to construct a 0–100 scale, with a higher score indicating better health [17].

### Employment Participation

Employment participation was measured with two questions: “Are you currently participating in paid employment?”. If this was the case, a follow-up question was asked: “How many hours per week are you participating in paid employment?”.

### Work Ability

Work ability was measured with a single question of the Work Ability Index, known as the Work Ability Score (WAI 1), with a 0–10 point Likert scale answer, with “If you give your work ability in the best period of your life 10 points, how many points would you give your work ability at this moment?”. Participants could then answer on a 0–10 point Likert scale answer. The Work Ability Index was reduced to a single question to minimize the burden on participants, as done in previous research [18–20].

### Per Protocol: Exercise Works

A participation rate was calculated based on the participant’s attendance in each session, divided by the total amount of sessions. The participation rate was split into at least 70% attendance compared to less than 70% attendance for a per protocol analysis. The varying duration and intensity of the program was accounted for in this split calculation. With this participation rate, a per-protocol analysis was performed.

### Individual Characteristics

Information on age, sex, migration background, marital status, having children, and highest obtained form of education were collected at baseline. Migration background was dichotomized into being born in the Netherlands or in another country. Marital status was divided into being married or living with a partner or living alone. The highest obtained form of education was divided into primary (pre-primary, primary, and lower-secondary), secondary (upper-secondary), and tertiary (post-secondary and tertiary) education based on the International Standard Classification of Education [21].

### Process Evaluation

Interviews were performed between June 2023 and January 2024. The goal of the interviews was to gain insight in the barriers and facilitators of the Exercise Works program. In total, 11 coaches of Exercise Works, 20 participants, and 10 employment professionals were interviewed. The interviews were conducted in person or through Microsoft Teams, depending on the preference of the interviewee. All interviews were audio recorded and transcribed verbatim. Afterward, the transcripts were analyzed in NVIVO version 1.7. The interviews were coded by the first author. Doubts or unclarities were discussed with the other authors. Coding was performed based on a thematic analysis. After coding the interviews, the codes were individually analyzed to structure recurring themes and differences.

### Statistical Analysis

Firstly, descriptive statistics were performed to describe the socio-demographic and household characteristics of the study population. Secondly, descriptives were used to investigate differences between the control- and intervention in employment, work ability, mental and physical health at baseline, 3 months, and at 12 months. These analyses were performed in SPSS version 28.

Thirdly, mixed effects linear regression analyses were performed to investigate the changes within individuals in employment participation, work ability, physical and mental health over the study period. The following model was used:

$${\mathrm{Y}}_{{{\mathrm{it}}}} = \, \beta_{0} + \, \beta_{{1}} \left( {{\mathrm{time}}} \right) \, + \, \beta_{{2}} \left( {{\mathrm{intervention}}} \right) \, + \, \beta_{{3}} \left( {{\mathrm{Intervention}}*{\mathrm{time}}} \right) \, + {\text{ e}}_{{\mathrm{t}}}$$

here, Y_it_ is the outcome variable (paid employment/work ability/physical health/mental health) of a person i at time t. Time is a continuous variable starting at baseline until the time of completion of the last questionnaire. Intervention is a dichotomous variable indicating the intervention or control group. In this model, β_1_ estimates the change in the outcome measures over time within the selected group; β_2_ estimates the difference at baseline between the Exercise Works and the control group; and β_3_ estimates the difference in the change over time between the Exercise Works and the control group. A random intercept was included to account for i) individual differences in the baseline level of the outcome variable, and ii) the correlation of the repeated measurements within the same individual. All analyses were adjusted for age, sex, educational level, and migration background. The Cohen’s d was also calculated to define the difference in effect size between the Exercise Works group and the control group. This was calculated by dividing the mean difference by the pooled standard deviation. Additionally, stratified analyses were performed based on sex, age, education level, and participation rate. These linear mixed models were performed in Stata version 18.

## Results

Table 1 describes the differences at baseline between the Exercise Works group and the control group. At baseline, Exercise Works participants were on average slightly older (mean 43.1 years) compared to the control group (40.0 years). Exercise Works participants were less often born in the Netherlands (64.9% compared to 84.6% in the control group). Additionally, Exercise Works participants completed tertiary education more often (10.1%) compared to the control group (4.3%).

**Table 1:** Socio-demographic characteristics at baseline of participants of the Exercise Works program (*n* = 208) and the control group (*n* = 117)

	Exercise works	Control group
Mean age (sd)	43.1 (11.5)*	40.0 (14.7)
	N (%)	N (%)
Female	125 (60.1)	78 (67.2)
Born in the Netherlands	135 (64.9)*	99 (83.9)
Married or living together	36 (17.3)	34 (29.1)
Children	78 (37.5)*	61 (52.1)
Education		
Primary	79 (38.0)*	64 (54.7)
Secondary	108 (51.9)*	48 (41.0)
Tertiary	21 (10.1)*	5 (4.3)

*Significant difference between the Exercise Works and the control group at *p* < 0.05

Table 2 shows that all outcome measures improved during the follow-up period of 12 months among participants of the Exercise Works program. The percentage of persons participating in employment for at least one hour per week increased from 2.4% at baseline to 28.1% after 12 months, whereas work ability increased from 4.3 to 5.4 on a scale from 1 to 10. Physical health increased from 42.8 at baseline to 48.5 after 12 months, whereas mental health increased from 47.1 to 52.4 after 12 months, both on a scale from 0 to 100. The control group showed mixed results, with employment participation steadily increasing, while physical and mental health increased before the second questionnaire and decreased thereafter.

**Table 2:** Employment, work ability, and health at baseline, after three months and after 12 months among participants of Exercise Works and the control group

	Exercise works			Control group		
	Baseline (*N* = 208)	3 months (*N* = 104)	12 months (*N* = 64)	Baseline (*N* = 117)	3 months (*N* = 71)	12 months (*N* = 61)
Employed N (%)	5 (2.4)	8 (7.7)	18 (28.1)	6 (5.0)	19 (25.3)	17 (25.8)
	Mean (sd)			Mean (sd)		
Work ability (1–10)	4.3 (2.5)	5.2 (2.5)	5.4 (2.6)	5.9 (3.0)	6.4 (2.8)	6.4 (2.6)
Physical health (0–100)	42.8 (14.1)	45.5 (11.7)	48.5 (14.6)	56.0 (24.4)	60.1 (21.5)	57.3 (22.5)
Mental health (0–100)	47.1 (16.8)	51.3 (19.2)	52.4 (19.5)	52.5 (19.7)	55.6 (19.6)	53.0 (22.2)

The results of the generalized linear mixed model showed an increase for all outcome measures among participants of Exercise Works. Physical health showed a yearly increase of 3.78 (95% CI 1.19;6.36), closely followed by mental health (2.82, 95% CI 0.08;5.56). Work ability increased with 0.69 (95% CI 0.27;1.10). However, these improvements did not differ significantly from the changes in the control group. The Cohen’s d ranged from -0.22 in employment participation to 0.13 for physical health (Table 3).

**Table 3:** Change over a one-year period in employment, work ability, and health among participants of Exercise Works and the control group

	Change in time β (95% CI)		
	Exercise Works	Control group	Difference
Employment	0.18 (0.13;0.24)*	0.22 (0.13;0.31)*	− 0.04 (− 0.14;0.06)
Work ability (1–10)	0.69 (0.27;1.10)*	0.39 (− 0.23;1.00)	0.30 (− 0.44;1.05)
Physical health (0–100)	3.78 (1.19;6.36)*	1.30 (− 2.52;5.11)	2.48 (− 2.13;7.08)
Mental health(0–100)	2.82 (0.08;5.56)*	1.00 (− 3.05;5.04)	1.83 (− 3.06;6.71)

Adjusted for age, sex, migration background, and education level

*significant at *p* < 0.05

Among the 84 participants who participated at least 70% of the program, stronger improvements were observed in physical health (5.68, 95% CI 1.97;9.40), closely followed by mental health (4.23, 95% CI 0.51;7.94) compared to those who participated less than 70% of the program. Larger effect sizes were seen among those who participated at least 70% (Cohen’s D: − 0.05 to 0.20), compared to those who participated less (Cohen’s D: − 0.32 to 0.08) (Table 4).

**Table 4:** Change over a one-year period in employment, work ability, and health among participants of Exercise Works and the control group, stratified on participation rate in program sessions of at least 70%

	Change in time with a participation rate of at least 70% β (95% CI)		
	Exercise Works	Control group	Difference
Employment	0.22 (0.13;0.30)*	0.23 (0.25;0.32)*	− 0.01 (− 0.14;0.32)
Work ability (1–10)	0.72 (0.14;1.31)*	0.39 (− 0.24;1.03)	0.33 (− 0.53;1.19)
Physical health (0–100)	5.68 (1.97;9.40)*	1.21 (− 2.82;5.25)	4.47 (− 1.02;9.95)
Mental health (0–100)	4.23 (0.51;7.94)*	0.93 (− 3.10;4.96)	3.29 (− 2.19;8.77)

	Change in time with a participation rate of less than 70% β (95% CI)		
Employment	0.17 (0.08;0.26)*	0.22 (0.13;0.31)*	− 0.05 (− 0.18;0.08)
Work ability (1–10)	0.63 (0.01;1.25)*	0.38 (− 0.21;0.97)	0.26 (− 0.60;1.11)
Physical health (0–100)	1.48 (− 2.63;5.58)	1.17 (− 2.73;5.07)	0.31 (− 5.35;5.96)
Mental health (0–100)	2.41 (− 2.01;6.82)	0.96 (− 3.24;5.17)	1.44 (4.64;7.54)

Adjusted for age, sex, migration background and education level

*Significant at *p* < 0.05

Among female participants of the Exercise Works program, a profound increase in mental (5.06, 95% CI 1.68; 8.43) and physical (4.62, 95% CI 1.29;7.95) health was seen, although this change was not statistically different from the females of the control group (Supplementary Table 1). Among participants of the Exercise Works program aged 40 years or younger, a significant improvement in work ability (1.11, 95% CI 0.05;2.16) was found in comparison with the control group. While mental health showed a non-significant change (6.97, 95% CI − 0.94;14.87) between Exercise Works participants younger than 40 compared to the same aged participants of the control group, the Cohen’s d illustrated a moderate effect (0.39) (Supplementary Table 2). Exercise Works participants with an intermediate education level showed a significant increase in mental health (7.87, 95% CI 0.87; 14.87, Cohen’s d 0.46) compared to the control group. Although the change in work ability among participants with an advanced education level was not statistically different between the two groups (1.52, 95% CI -1.11; 4.14), the Cohen’s d (0.61) did show an effect of moderate magnitude (Supplementary Table 3).

The results from the interviews showed that interviewees were very positive about participating in the Exercise Works program. Participants mentioned that they greatly enjoyed the way the lifestyle education was taught and the playful approach to physical activities. Exercise Works coaches emphasized the value of seeing participants twice a week and having a low entrée threshold. The coaches mentioned that, due to the current high demand for workers in the labor market, non-employed individuals enrolled in the Exercise Works program often faced complex problems that substantially hampered their capability to return to the labor market. This was also mentioned by the employment services. However, some professionals stated that even if progress during the program was limited, it was still of great value to gain insight into the capabilities and resilience of participants.

Participants in the Exercise Works program expressed a very positive attitude toward the program. They recommend it to others in their social circles. The key elements they highlight are that they are motivated to change in a playful manner, that they meet new people, and that they engage in physical activity together. It is particularly due to the social influence within the group that participants report being more willing to engage in activities they initially were not inclined to do. Additionally, information is conveyed in an accessible manner, and participants are provided with a daily rhythm and routine. Most participants indicate that they enjoyed the sports and games sessions the most, particularly when they take place outdoors. The participants suggested that the amount of outdoor sports should be increased. In municipalities where volunteer work is also part of the program's exit opportunities, participants expressed great appreciation for this aspect. Furthermore, they noted that the program requires significant time commitment and often early starting times. Some participants would prefer to start later in the day. Overall, the program meets the participants' expectations, although most indicated that this was due to starting with few expectations (Table 5).

**Table 5:** Quotes from the interviews

Exercise Works coach 1	But now, with the group of 14: 'Hey, where are those two?' And if necessary, we'll pick them up next time, so to speak, because they’re missing out on the fun, and that’s the feeling I get now. So you really see the effect of group dynamics."
Employment professional 1	So, as an employment professional, it just gives insight into how much more you get when you see someone twice a week. If I only spoke to someone once a week, I’d have them in my office for a bit or go for a walk, but two mornings a week gives you a really good sense of who someone is, where their boundaries are. So it always results in meaningful insights. Even if someone turns out to not be able to do anything, that becomes clear too."
Employment professional 4	"When someone says, 'My Dutch isn’t very good,' and you ask, 'Would you like to take a course?' And they say, 'No, that's not necessary.' But then they’re in a group, and someone says, 'Hey, why don’t you come with me to Dutch class, your Dutch could be better.' And then they go ahead and do it. Or they join someone for volunteer work, ‘It’s fun, come with me and check it out.’ So you’re making use of it in two ways Sometimes there are small groups of people who even go for walks together in the park. And it extends to their families, like when they want to lose weight, and then the whole family starts eating healthily. So there is so much more involved."
Participant 7	I move more at home now. One of my goals is also to go out with my kids and do more sports. My goal is to enjoy sports more. And I had rollerblades, but they were no longer in good condition, so I bought new rollerblades to go rollerblading with my daughter."
Participant 13	I feel better, also about myself, my self-confidence has gone up. What else? I’m more mindful of the choices I make. Instead of gaming, I now go for a walk. So I pause more and think about what I can do to reach my goals, which helps a lot. And other than that, well, as I said, the emotional regulation part—I feel a bit more neutral. I still have occasional mood swings, but I feel more neutral overall, so the exercise is really working
Participant 19	Yes, exercise works, moving a lot. So yes, I do that. And the advantage is that you can also go to the Exercise Works Coach for other things, so that’s really the bonus with exercise works. The mental aspect, which I’m really struggling with at the moment, I can also go to the Exercise Works Coach for that. Fortunately, I can also turn to other people because otherwise the Exercise Works Coach would go crazy with 15 participants per round

## Discussion

This study demonstrated no differences in improvements in physical or mental health, workability, and employment participation over time when comparing the Exercise Works group with the control group. Modest improvements in physical and mental health, workability, and employment participation among participants of Exercise Works were shown. The control group also showed some improvements, albeit of lesser magnitude. The subgroup analyses showed significant benefits in work ability among Exercise Works participants younger than 40 years and in mental health among participants with an intermediate education, compared to the control group. Participants who attended at least 70% of the time showed stronger improvements compared to participants who attended less.

The general improvements among Exercise Works participants were not stronger compared to participants of the control group. This is in line with the earlier research performed on Exercise Works in 2009. However, the current study did detect a greater improvement in physical and mental health [14]. A recent meta-analysis found that health promotion programs for non-employed individuals only had small effects on mental health. However, the authors did conclude that even though the effect sizes might be small, these health promotion programs could improve the health of many non-employed individuals [12]. This is in line with the current study, where improvements of small magnitude were found in the Exercise Works group, which were not larger compared to the control group.

During the current study period, the unemployment rate was 3.5% to 4.2%, whereas in the previous study during 2008–2009, unemployment rates were 4.8% to 5.4% [22]. Hence, in the current study, the labor market offered more opportunities for paid employment. This means that for individuals searching for employment, it is relatively easy to find a suitable job. Clients with any employability are already guided to employment, leaving clients with more complex problems behind. The latter hardcore group is then enrolled into the Exercise Works program. This creates difficulties during the program, since these complex problems require more attention and often a more personalized approach. The selection of persons with worse health into long-term unemployment is shown upon comparing baseline physical and mental health of this study to previous studies. Physical health was 10 points lower (42.8 vs. 52.8) and mental health 5 points lower (47.1 vs. 52.5) in the current study compared to the previous study on an earlier version of the intervention [14].

This study shows a discrepancy between the interviews and the questionnaire results. The changes in health- and employment outcomes among participants of the Exercise Work group are not statistically significant compared to the control group. Based on the interviews, everyone involved was delighted with the program. Participants were really enthusiastic about the program and would recommend it to their peers. However, social desirability bias and selection bias might have occurred. Only 10% of the participants were interviewed and it was not always possible to perform the interviews in an enclosed space, sometimes the coach would walk past during the interview. This might have created an environment for participants to answer socially desirable [23]. Employment professionals described it as ‘a present for the participant.’ Even if a client dropped out or was not able to enter paid employment after the program, employment professionals described it as valuable information on the resilience and capacities of the client. Only few barriers were found on the execution or implementation of the program. This included difficulties in renting a location, a high workload and for smaller municipalities it is more difficult to recruit enough participants.

Additionally, part of this research was conducted during the COVID-19 pandemic. This not only affected the execution of the program and the research, but also the wellbeing of participants. Due to the measures put in place by the government, the program was not allowed to take place or had to be executed in small groups, outside or only inside with the availability of a QR code of a COVID vaccination or test. Because of this, the research was postponed and included less participants, which contributed to the reduced power of the study. Lastly, a large loss to follow-up was seen among participants of the Exercise Works program. This could be due to other problems in their life (e.g., health, financial problems) or them not being enthusiastic about the program. In order to increase the completion rate of the last questionnaire, an incentive was created by sending out gift cards if a participant completed the questionnaire. However, a large dropout still remained. Future research should adopt more intensive strategies create a focus on how to decrease the dropout rate. Since there was a discrepancy between the results obtained from the questionnaires and the interviews, future research is needed to better understand the underlying factors contributing to this discrepancy. Future studies should focus on developing a realistic evaluation approach that considers both quantitative and qualitative data, to ensure a better understanding of the program’s effectiveness.

In conclusion, this study showed no significant improvements in health- and employment outcomes among participants of the Exercise Works program. Both participants and professionals had a positive perception of the program. It remains important to include individuals with complex problems in health promotion and re-employment programs, in order for them to participate in society. These complex problems should be taken into account when developing a health promotion program for this vulnerable group.

## Supplementary Information

Below is the link to the electronic supplementary material.

Supplementary file1 (DOCX 45 KB)

## Data Availability

The datasets generated during the current study are not openly available.
