# Supplementary material for: The Influence of a Health Promotion Program on Health and Paid Employment Among Long-Term Non-employed Individuals in the Netherlands
Source: J Occup Rehabil. 2025 May 9;36(2):493–502. doi: 10.1007/s10926-025-10290-7 (PMC13099847; doi:10.1007/s10926-025-10290-7)
Supplement: Supplementary file 1 — Supplementary file1 (DOCX 45 KB) [file 10926_2025_10290_MOESM1_ESM.docx]

# Supplementary file

Supplementary table 1 Employment, health and lifestyle determinants among persons who participate in the Exercise Works program or the control group at baseline and change in months over one year time, stratified on sex

|  | Change in time  β (95% CI) | | | Cohen’s D |
| --- | --- | --- | --- | --- |
|  | Exercise Works | Control group | Difference |  |
| Male (N=122) | | | |  |
| Employment | 0.16 (0.07;0.26)* | 0.44 (0.27;0.61)* | -0.28 (-0.47;0.08) | -1.79 |
| Work ability (1-10) | 0.57 (-0.21;1.34) | 0.77 (-0.59;2.14) | -0.21 (-1.7;1.36) | -0.08 |
| Physical health (0-100) | 2.31 (-1.67;6.29) | 1.64 (-5.27;8.55) | 0.67 (-7.29;8.64) | 0.04 |
| Mental health(0-100) | -1.19 (5.80;3.42) | -0.22 (-8.24;7.81) | -0.97 (-10.21;8.26) | -0.05 |
| Female (N=203) | | | |  |
| Employment | 0.19 (0.13;0.26)* | 0.14 (0.05;0.24)* | 0.05 (-0.07;0.17) | 0.26 |
| Work ability (1-10) | 0.80 (0.31;1.28)* | 0.31 (0.36;0.98) | 0.49 (-0.34;1.31) | 0.18 |
| Physical health (0-100) | 4.62 (1.29;7.95)* | 0.97 (-3.62;5.56) | 3.65 (-2.02;9.33) | 0.18 |
| Mental health(0-100) | 5.06 (1.68;8.43)* | 1.19 (-3.45;5.83) | 3.87 (-1.87;9.60) | 0.21 |

Adjusted for age, migration background and education level. * Significant at p < 0.05

Supplementary table 2 Employment, health and lifestyle determinants among persons who participate in the Exercise Works program or the control group at baseline and change in months over one year time, stratified on age

|  | Change in time  β (95% CI) | | | Cohen’s d |
| --- | --- | --- | --- | --- |
|  | Exercise Works | Control group | Difference |  |
| Younger than 40 years (N=144) | | | |  |
| Employment | 0.26 (0.15;0.36)* | 0.31 (0.17;0.46)* | -0.06 (-0.23;0.12) | -0.32 |
| Work ability (1-10) | 1.39 (0.78;2.01)* | 0.29 (-0.58;1.15) | 1.11 (0.05;2.16)* | 0.39 |
| Physical health (0-100) | 5.95 (1.90;10.01)* | 2.70 (-3.03;8.43) | 3.25 (-3.76;10.25) | 0.18 |
| Mental health(0-100) | 6.01 (1.43;10.59)* | -0.96 (-7.42;5.51) | 6.97 (-0.94;14.87) | 0.39 |
| 40 years and older (N=191) | | | |  |
| Employment | 0.13 (0.06;0.19)* | 0.15 (0.05;0.25)* | -0.02 (-0.14;0.10) | -0.11 |
| Work ability (1-10) | 0.18 (-0.38;0.73) | 0.47 (-0.38;1.32) | -0.29 (-1.30;0.73) | -0.11 |
| Physical health (0-100) | 2.44 (-0.90;5.77) | -0.01 (-5.10;5.09) | 2.44 (-3.65;8.53) | 0.12 |
| Mental health(0-100) | 0.75 (-2.59;4.10) | 2.59 (-2.51;7.70) | -1.84 (-7.94;4.26) | -0.10 |

Adjusted for sex, migration background and education level. * Significant at p < 0.05

Supplementary table 3 Employment, health and lifestyle determinants among persons who participate in the Exercise Works program or the control group at baseline and change in months over one year time, stratified on education level

|  | Change in time  β (95% CI) | | | Cohen’s d |
| --- | --- | --- | --- | --- |
|  | Exercise Works | Control group | Difference |  |
| Basic education (N=143) | | | |  |
| Employment | 0.10 (0.01;0.19)* | 0.31 (0.19;0.43)* | -0.20 (-0.35;0.05)* | -1.21 |
| Work ability (1-10) | 0.83 (0.10;1.56)* | 0.37 (-0.54;1.29) | 0.46 (-0.72;1.63) | 0.16 |
| Physical health (0-100) | 2.61 (-2.06;7.29) | 2.25 (-3.59;8.08) | 0.37 (-7.11;7.84) | 0.02 |
| Mental health(0-100) | 0.82 (-3.94;5.58) | 4.02 (-1.87;9.92) | -3.21 (-10.78;4.37) | -0.17 |
| Intermediate education (N=156) | | | |  |
| Employment | 0.18 (0.11;0.26)* | 0.11 (-0.01;0.23) | 0.07 (-0.07;0.21) | 0.34 |
| Work ability (1-10) | 0.40 (-0.16;0.95) | 0.32 (-0.55;1.19) | 0.07 (-0.96;1.11) | 0.03 |
| Physical health (0-100) | 4.43 (1.07;7.79)* | -0.82 (-6.13;4.50) | 5.24 (-1.04;11.53) | 0.27 |
| Mental health(0-100) | 4.55 (0.81;8.29)* | -3.32 (-9.25;2.61) | 7.87 (0.87;14.87)* | 0.46 |
| Advanced education (N=26) | | | |  |
| Employment | 0.44 (0.28;0.60)* | 0.42 (-0.02;0.85) | 0.02 (-0.45;0.49) | - |
| Work ability (1-10) | 1.86 (0.95;2.78)* | 0.35 (-2.11;2.80) | 1.52 (-1.11;4.14) | 0.61 |
| Physical health (0-100) | 5.41 (-0.63;11.44) | 15.18 (-0.94;31.29) | -9.77 (-27.02;7.48) | -0.63 |
| Mental health(0-100) | 0.58 (-4.87;60.3) | 15.84 (1.33;30.35)* | -15.26 (-30.78;0.26) | -0.70 |

Adjusted for age, sex and migration background * Significant at p < 0.05
